# The political economy of results-based financing: the experience of the health system in Zimbabwe

**DOI:** 10.1186/s41256-019-0111-5

**Published:** 2019-07-15

**Authors:** Sophie Witter, Yotamu Chirwa, Pamela Chandiwana, Shungu Munyati, Mildred Pepukai, Maria Paola Bertone

**Affiliations:** 1 ReBUILD programme, Queen Margaret University, Edinburgh, EH21 6UU UK; 2 ReBUILD and Biomedical Research and Training Institute, P O Box CY 1753, Harare, Zimbabwe; 3 Biomedical Research and Training Institute, P O Box CY 1753, Harare, Zimbabwe

**Keywords:** Political economy analysis, Results-based financing, Zimbabwe, Health financing reforms, Fragile and crisis-affected settings

## Abstract

**Background:**

Since 2000, results based financing (RBF) has proliferated in health sectors in Africa in particular, including in fragile and conflict affected settings (FCAS) and there is a growing but still contested literature about its relevance and effectiveness. Less examined are the political economy factors behind the adoption of the RBF policy, as well as the shifts in influence and resources which RBF may bring about. In this article, we examine these two topics, focusing on Zimbabwe, which has rolled out RBF nationwide in the health system since 2011, with external support.

**Methods:**

The study uses an adapted political economy framework, integrating data from 40 semi-structured interviews with local, national and international experts in 2018 and thematic analysis of 60 policy documents covering the decade between 2008 and 2018.

**Results:**

Our findings highlight the role of donors in initiating the RBF policy, but also how the Zimbabwe health system was able to adapt the model to suit its particular circumstances – seeking to maintain a systemic approach, and avoiding fragmentation. Although Zimbabwe was highly resource dependent after the political-economic crisis of the 2000s, it retained managerial and professional capacity, which distinguishes it from many other FCAS settings. This active adaptation has engendered national ownership over time, despite initial resistance to the RBF model and despite the complexity of RBF, which creates dependence on external technical support. Adoption was also aided by ideological retro-fitting into an earlier government performance management policy. The main beneficiaries of RBF were frontline providers, who gained small but critical additional resources, but subject to high degrees of control and sanctions.

**Conclusions:**

This study highlights resource-seeking motivations for adopting RBF in some low and middle income settings, especially fragile ones, but also the potential for local health system actors to shape and adapt RBF to suit their needs in some circumstances. This means less structural disruption in the health system and it increases the likelihood of an integrated approach and sustainability. We highlight the mix of autonomy and control which RBF can bring for frontline providers and argue for clearer understanding of the role that RBF commonly plays in these settings.

## Background

Over the last decade, results based financing (RBF) has been increasingly implemented in low- and middle-income countries to purchase health care services. These schemes, often also called performance based financing, typically aim to improve health services by providing bonuses to service providers (usually facilities, but often with a portion paid to individual staff) based on the verified quantity of outputs produced, modified by quality indicators. In many cases there is a division of functions between regulation, purchasing, fund-holding, and service delivery [1]. While research and evidence on RBF has grown since the first systematic review [2], much of the literature has focused on the health outcomes and outputs of RBF [3–6], while less attention has been paid to the drivers of policy and of how RBF is implemented and rolled out under different conditions and settings. This is important as there is controversy about effectiveness and the health system effects of RBF programmes, as well as the extent to which they are driven by external agendas [7]. There is also evidence that RBF programmes are more common in fragile and conflict-affected settings and that this may relate to the greater role of external actors [8]. However, there are still few case studies exploring these dynamics, in particular based on FCAS experiences. Moreover, existing studies have adopted a focus on agenda-setting and scale-up in RBF programmes, rather than an explicit political economy framing [9–12]. One of the factors which has limited study in this area is likely to be its contentious nature, particularly for those with a stake in the RBF sphere.

In this article we examine the political economy of RBF in Zimbabwe, asking, first, how historical legacies, ideological values and framing influenced its adoption; secondly, how roles, decision-making and power relationships played out in relation to RBF’s later implementation and evolution; and, thirdly, how RBF has (or has not) shifted power and resources in the Zimbabwe health system. The overall objective is to understand the influence of the starting context, institutions and actors on the programme’s development and adoption, and, in turn, how the programme shaped these factors. The case study is illuminating as Zimbabwe is one of a small number of African countries where the programme is operating on a national scale, and understanding its overall evolution can produce important lessons for funders and countries engaged in or contemplating introducing RBF, as well as for the wider research community. Political economy analysis is recognized as an important tool to examine how formal and informal institutions influence and are influenced by decision-making, power and resources [13]. This article extends its application by applying it to a health financing reform in a low-income, fragile setting.

## Methods

### Study design

The case study is largely retrospective qualitative case study, focusing on the period since 2011, although drawing on insights into the health system in Zimbabwe from earlier studies [14, 15]. It is based on key informant interviews at national, provincial and district levels, integrated with analysis of documentation (policies and strategies, project documents and manuals, project evaluations, academic articles). The research team also drew on their knowledge of the policy environment.

The study is organised around a political economy analysis (PEA) framework, which is suited to examining the interaction of actors, context and resources (such as power and financing). Table 1 presents the PEA themes, which informed the questions which were used in the topic guide, and were also used for analysis of interviews and documents. These were adapted from a prior political economy framework [16], with questions on framing and recommendations added. The framework was chosen as it is designed for broad analysis of sectoral policies and probes the key variables of interest to this study – the influence of institutions and actors’ positioning on the RBF programme, and in return, how it affects their position. A set of questions on contextual factors – particularly, historical legacies, ideologies and values, and the framing of the RBF concept – explore how these influenced the adoption of RBF (our first question). A second set, more focused on actors, decision-making and roles, explores RBF’s implementation (our second question). Finally, we explore how RBF affected the distribution of resources in the sector (our third question) – specifically, which actors gained or lost from its introduction, and how it impacted on health financing, equity and corruption. As the research area was relatively new, the aim was exploratory, rather than starting from specific hypotheses.

**Table 1:** Political economy themes used in study

Domain	Topic	Questions posed
Context	Historical legacies	What is the past history of the sector, including previous health reform initiatives and experience of crisis? How do these influence current stakeholder perceptions? How far did RBF respond to or reflect these historical legacies?
	Ideologies and values	What are the dominant ideologies and values which shape views around the health sector? To what extent did these influence the adoption of RBF? How have they been altered by it?
	Framing of concept	How was RBF portrayed by key stakeholders in the adoption phase? How did this framing change over time? What is the dominant narrative behind RBF? Is there a consensus or disagreement on what it means or how it is meant to work?
Actors	Decision making	How are decisions made within the health system in Zimbabwe? Who is party to these decision-making processes? What role does evidence play? How are trade-offs managed? How were these reflected in the adoption, adaption and implementation of RBF? Has RBF influenced these processes in turn?
	Roles and power relationships	Who are the key stakeholders in the health sector in Zimbabwe? What are the formal/informal roles and mandates of different players? What is the balance between players at different levels of the health system? To what extent is power vested in the hands of specific individuals or groups in relation to the health sector in Zimbabwe? How do different interest groups outside government (e.g. private sector, non-governmental organisations, consumer groups, the media) seek to influence policy? How were these reflected in the adoption, adaption and implementation of RBF? Haw has RBF influenced these roles in turn?
	Donor roles and coordination	What role have external development partners played in relation to the health system in Zimbabwe? How well do they cooperate and coordinate? Do they always support national priorities? Do you think they have their own political economy incentives to pursue particular approaches? How were these reflected in the adoption, adaption and implementation of RBF? Has RBF influenced these processes in turn?
Distribution of resources	Support for reform	Who were the “winners” and “losers” from RBF, at different stages? Who are its key champions? How much political priority does RBF have and why? Who is resisting, and why?
	Ownership structure and financing	How is the sector financed? What is the balance between public and private ownership? How did these feed into the adoption, adaption and implementation of RBF? How has RBF in turn influenced ownership and financing in the sector?
	Corruption and rent seeking	Is there significant corruption and rent-seeking in the health sector? Where is this most prevalent (e.g. at point of delivery; procurement; allocation of jobs)? Who benefits most from this? How is patronage being used? How were these reflected in the adoption, adaption and implementation of RBF? Has RBF influenced these processes in turn?
	Service delivery	Who are the primary beneficiaries of service-delivery? Are particular social, regional or ethnic groups included or excluded? How are subsidies provided, and which groups benefit most from these? How were these reflected in the adoption, adaption and implementation of RBF? Has RBF influenced these equity outcomes in turn?
Institution-alisation	Recommendations	In order to make RBF effective and sustainable in this context in future, what would you recommend?

### Study sites

Data collection was done at national level and in two provinces (Midlands and Mashonaland East), including four districts (Murewa, Marondera, Gokwe North and Gokwe South). These provinces were selected as they were the site of the pilot districts for the first RBF scheme in 2011. The districts were chosen as representing one each from the two RBF schemes per province (the World Bank programme and the Health Development Fund (pooled donor)-supported RBF scheme) and including the original pilot sites to gain the longest lens possible on the RBF adoption and roll out.

### Document review

We searched for documents on RBF in Zimbabwe and extracted data, using the political economy analysis framework [16].

Documentary sources included the World-Bank RBF website, the RBF Community of Practice, government’s websites, key informants from government departments, donors and non-governmental organisations, including the implementers in Zimbabwe, Cordaid and Crown Agents, as well as documents already available to the team through long term engagement in-country of the researchers. The documents included the following:

- National Health Strategic Plans and health financing policy documents
- RBF implementation/operational manuals
- RBF evaluations and reports
- Minutes and reports from meeting and working group discussions
- Academic articles
- Datasets on RBF bonus payments and results

A snowball technique was adopted by checking the references provided in the documents analysed and retrieving further relevant documents. Similarly, during key informant interviews, interviewees were asked if they could share other relevant documentation.

The documents date from the decade from 2008—i.e. after the most acute period of crisis and prior to the introduction of RBF in 2011—up till 2018. Some 60 documents were reviewed, the vast majority of which were operational and grey literature.

### Key informant interviews

Purposive sampling was used to identify key informants (KI) at national, provincial and district levels, based on their knowledge and involvement on RBF from its inception till the present. The selection of interviewees was as comprehensive as possible, including individuals currently holding RBF-related posts or who were previously in such positions. A number of relevant organizations, groups and individuals involved in RBF were preliminarily identified. New individuals were added based on the results of the documentary review or as suggested by key informants. Interviewees included representatives of:

- Ministry of Health and Child Care (MoHCC): Departments at central level, but also Provincial Medical Directors, Provincial Health Executives and District Health Executives (DHEs)
- Other relevant ministries and national organisations, including the Ministry of Local Government and Rural Development and the Zimbabwe Association of Church-related Hospitals
- Donors/funders present and past: World Bank, Health Development Fund (HDF) partners, the UK Department for International Development and European Union
- RBF implementers: Cordaid and Crown Agents (two international non-governmental organisations), at central and district levels; UNICEF (fund manager for the HDF)
- Consultants and technical assistants who had worked on RBF

The breakdown of key informants interviewed (40 in total) is provided in Table 2. Eighteen MoHCC staff were interviewed at national, provincial and district levels. The development partner group was the next-largest constituency, with 10 key informants. Overall, men predominated, reflecting gender discrepancies, particularly at higher levels of the health system. For RBF implementers, by contrast, staff at central and field offices were more commonly female.

**Table 2:** Key informants summary

	Male	Female	Total
National MoHCC staff	5	0	5
Provincial health executives	3	3	6
District health executives	4	3	7
Other ministries and public bodies	1	2	3
Development partners	7	3	10
Consultants	3	0	3
Implementers	1	5	6
TOTAL	24	16	40

Key informants were approached by email or telephone, providing them with a brief explanation of the research project. A time and date for an interview was agreed upon. Before the interview, the researcher explained the study objectives and scope, and informed consent was obtained in writing. Confidentiality was assured. Consent was requested for recording, with manual note-taking as a fall-back option where the respondent was not comfortable with the conversation being recorded or where security arrangements or technology did not permit recording. Twenty-six out of 40 interviews were recorded.

Key informants were interviewed in English, using a semi-structured interview guide, structured around the political economy analysis framework [16]. Most interviews took place in the informant’s place of work, but in a location where privacy was assured. Some interviews were conducted by phone or Skype, where physical distance or access necessitated it. Interviews focused on the period from 2008 (prior to RBF introduction) to present and were tailored to the time available and the knowledge of the KI. Interviews lasted from 30 min to 2 h, with an average of 1 h. The questioning was led by a senior researcher, with a colleague assisting in taking notes. Interviews took place from early February to late March 2018.

### Data analysis

Data analysis was done iteratively. A first thematic analysis based on the PEA framework of the documents collected was conducted before the interviews in the field, and guided the discussion during interviews. Later on, new documents were added to the review, and a final thematic analysis [17] was conducted of documents and interview transcriptions or notes. Results of the analysis of documents and interviews were written-up together to allow for triangulation and complementarity between data sources. Some quotations are provided below to give texture and substance to key points, although space does not permit every point to be illustrated. All national and international KI are described in the results as ‘national KI’ (working at national level), while those from province, district and field offices are described as ‘local KI’. This amalgamation of categories was intended to protect anonymity, given the small pool of potential participants.

### Ethics

Ethical clearance was obtained from the Research Ethics Panel of Queen Margaret University, Edinburgh, and from the Medical Research Council of Zimbabwe. The study also received authorisation from the MoHCC.

## Results

We start by presenting a brief overview of the policy timeline and basic features of RBF design to frame the results (Table 3). We then present results by nine political economy themes adapted from the framework, grouped into three clusters. We start with contextual factors including the historical legacy and its impact on the adoption of RBF, and how ideologies and values influenced its adoption, evolution and framing. Next we focus on the role of actors—their roles, power relations and influence on the RBF decision-making. We then examine the how RBF has influenced the distribution of resources in the health system. Finally, we include some reflections to look to the future in relation to plans to institutionalise RBF in Zimbabwe, incorporating some of the recommendations of the KI.

**Table 3:** Timeline for RBF adoption, piloting and scale up

Date	Key events
2008	Peak of political/economic crisis in Zimbabwe
2009	Government of National Unity (GNU) takes power. 2009–13 National Health Strategy lays out plan for post-crisis recovery
2008–10	World Bank engages the Ministry of Health and Child Care (MoHCC) in discussion of RBF
July 2011	Pilots start in two ‘front-runner’ districts. National RBF steering committee established.
January 2012	The Health Transition Fund (HTF) provides pooled donor support to maternal, newborn and child health and health system strengthening – paying allowances to key staff, purchasing essential drugs and equipment, fixed amounts to rural health centres (RHCs) ($750 per quarter per clinic) across the country
March 2012	RBF pilot scaled up to 18 districts (two per province), managed by Cordaid and funded by the World Bank. First programme implementation manual (PIM) developed. Indicators focused on maternal and child services in primary clinics (public and mission sectors) but with support to referral facilities for specific indicators.
	HTF continues to support all districts with equipment, medicines and retention allowances but stops financial support to RBF clinics (fixed allowance continues to remaining 42 districts).
2012	World Bank Technical Review undertaken; first RBF price adjustment
2013	Mid-term review of Cordaid programme; prices adjusted for some indicators; quality bonuses shift to threshold-based system; greater weight given to clinical quality in quality checklist
2014	HTF adopts output-based model in 42 districts for primary care units only (because of resource constraints, district hospitals continue to receive fixed amounts in the 42 districts). UNICEF launches tender for implementation, which Crown Agent wins. Cordaid shares its model and helps to train staff in the 42 new districts.
2016	Impact evaluation of RBF in 18 districts shared^23, 52^. Zimbabwe hosts global RBF workshop, with World Bank support. Quality checklist revised.
	HTF transitions to Health Development Fund (HDF).
2017	Review of indicators and quantity/quality weighting, following health system assessment (focusing on RBF, human resources and pharmaceuticals). Staff bonus is linked to quality scores. Technical working group for sustainability established. Prices for indicators drop due to budget constraints.
November 2017	Political upheaval and start of ‘new dispensation’ led by Emmerson Mnangagwa.
2018	Institutionalisation plan aims to shift functions from external contractors to the Project Coordination Unit in MoHCC for 18 districts in 2018. MoF contribution to funding increased. District hospitals to be included in RBF programme for all districts.

### Policy timeline and RBF design

RBF started in July 2011, initially in two front-runner districts of Marondera and Zvishavane, then in an additional 16 districts, with funding from the World Bank and implemented by Cordaid (Table 3). The RBF programme pays for 16 indicators at primary health unit (PHU) level and five at referral level [18], with additional payments based on quality scores. It is focused on rural areas, covering all 60 rural districts and two urban districts [18], with the cities of Harare and Bulawayo excluded. This was scaled up to all rural facilities in 2014, with funding for the other districts from the pooled Health Transition Fund (HTF), a multi-donor fund established in 2011 to support the Government of Zimbabwe to reduce maternal and child mortality, which later became the Health Development Fund (HDF). This RBF programme was implemented by Crown Agents, with funds managed by UNICEF.

### Context

#### Historical legacies

The context was key to the adoption of RBF in Zimbabwe. Zimbabwe experienced a severe economic and social crisis, which peaked in 2008 and is still on-going (see Table 4 on health care provision and financing in Zimbabwe). Zimbabwe’s dramatic economic collapse during the 2000s led to loss of skilled health personnel, lack of investment in health and chronic shortages of essential drugs and equipment [25]. Inadequate public financing resulted in introduction of formal and informal user charges that further restricted access to health care particularly for women and children. In 2008, the health system was close to collapse.

**Table 4:** Background on health care provision and financing in Zimbabwe

The public sector is the main provider of health care services [19]. Health care in Zimbabwe is delivered through 1848 facilities, most of which are public health care facilities (the largest category being the rural district council-run primary facilities). The rest are non-profit and church affiliated facilities (referred to as mission facilities), private for-profit facilities and company operated clinics^a^. Municipalities also fund and provide primary health care services in their areas. These health facilities are separate from those directly administered by the MoHCC. Local councils generate revenue through local taxes, levies and other fees. During the crisis, health financing collapsed, resulting in 0.02% of GDP in 2009 for MoHCC expenditure [20]. User fees picked up the gaps, rising from 23% in 1999 to 62% of total health expenditure in 2005^31^. Over the same period, health insurance payments are reported to have collapsed from 20% to less than 1 % of total health expenditure. However, by 2010, out of pocket payments had reduced to 39%, which was still well above the 20% maximum level prescribed by the World Health Organisation [21]. In 2015, government expenditure on health as a proportion of total government expenditure was approximately 8%, an increase on previous years but still low for the region [19]. Household payments accounted for around 25% of total health expenditures in 2015, of which 95% were out of pocket. In Zimbabwe, 7.6% of all households incurred catastrophic health payments in 2015; the incidence of catastrophic health payments was highest among households in the poorest quintile [19]. Donor funds provided roughly a quarter of total health expenditure, according to the 2015 national health accounts data. However, pooled funding comprised only 7% of donor funding while non-pooled funding made up the bulk of funding [22] and 90% of external aid to the sector came from ten partners, which creates risks [23]. Estimated costs for a medium-scenario package of care were $94 per capita in 2018, as against expected public resources of $52 per capita [23]. There is evidence of internal pressure for increased public commitments to health – Parliament held up the 2018 budget until the health allocation was increased - however, it remains low, with only one third of the amount needed by the sector funded [24], at around $27 per capita. Health’s allocation as a share of total government budget has remained within the range of 7–10% since 2010 [24].	

^a^There are 101 private health facilities and 87 mission facilities. Mission and private health facilities provide only primary and secondary care. Mission facilities are partly funded by the MoHCC through salary, administration and capital grants. 68% of services in rural areas and 35% nationally are delivered by mission facilities

Failing to take up of life saving interventions and poor quality of care contributed to the reversal of key health indicators. What had once been a well-functioning health system entered a period of rapid decline. Most external assistance focused support on disease specific interventions in particular against HIV/AIDS, tuberculosis and malaria. Yet Zimbabwe still had a residual skilled workforce, an underused health infrastructure and strong policy frameworks to improve maternal and child health and address underlying system weaknesses [26].

In 2009, a hundred day plan was developed to revitalise health services by the Government of National Unity. To mobilize resources, the Ministry developed and launched the Health Sector Investment Case in March 2010, which gave birth to the Health Transition Fund (HTF). The HTF was a multi-donor fund focused on strengthening the health system and ensuring access to health services in particular for pregnant women and children under five. HTF engaged to pay basic salaries throughout the country and ensure basic drugs supply and equipment. It aimed to revive what was described as a ‘*completely comatose health system at the time …*. *It was more profitable to sell tomatoes than work as a professional in health facilities at that time*’ (national KI).

In this context, the World Bank arrived with an offer of $15 million, which was conditional on using an RBF mechanism. As Zimbabwe was in arrears with its international debt payments, it was not eligible for regular International Development Association loans, and so the Health Results Innovation Trust Fund, which supports RBF approaches, was the only funding vehicle the World Bank could offer.

*‘There was no scope for negotiation as the Bank could only offer this kind of grant. The health system was very cash-strapped so it was hard for them to negotiate. There was only one offer on the table. It was a non-decision on both sides’* (national KI)

The Ministry and other donors were initially distrustful of the RBF approach, but there was an urgent need for resources. At this stage, the Ministry was deeply donor dependent. Many programme staff were funded by development partners. Planning meetings were described as ‘*just rituals*’ by one sub-national KI.

*‘We were very poor on donor coordination. This came when there was a crisis and no-one dared to tell them to sit down and do things properly’ (local KI).*

Despite the crisis, there remained a legacy of the pre-crisis stronger system and a resistance to a model which Zimbabweans saw as designed for countries with weak (or no) health systems. There was resistance to parallel programmes and confidence that with resources, the Zimbabwean health system could deliver. On this basis, the MoHCC opposed piloting and argued for quick scale up to two districts per province (in 2012).

*‘The World Bank wanted to pilot in two districts but we argued that the principles and ideas were not new to Zimbabwe. We needed resources and would work hard so it would definitely work’* (national KI)

This self-confidence, despite crisis and dependency, ensured that adaptations were made to the RBF model, which fitted it to the Zimbabwean context. Initially, the MoHCC resisted paying staff incentives as it felt these would undermine professionalism and might cause distortions in the labour market. However, given the low salaries, the need for staff motivation became clear and permission to allocate 25% of facility RBF payments to staff was given in 2013. The MoHCC also did not agree to giving the PHUs the power to hire and fire staff – which would have fitted with a ‘pure’ approach to provider autonomy within the RBF model, but hiring was seen as a centralised function and important to maintaining a systemic approach. The MoHCC placed great stress on the role of the Health Centre Committees (HCCs) and insisted on the need for district RBF steering committees in order to integrate RBF within wider planning and coordination mechanisms. One KI described their philosophy as ‘*guided democracy*’ (national KI) – in other words, achieving a balance between allowing facilities to set their own priorities and keeping a system with coordination and oversight.

As the World Bank funds could not go directly to the government, given the arrears with international institutions, Cordaid managed the RBF funds and modifications were made to allow government financial contributions to be paid to it as the implementing agency, which is very unusual for RBF schemes internationally. Public financial management rules also had to be changed to allow PHUs to open bank accounts. They had previously had virtual accounts at district level, with funds paid in from the Health Service Fund (a fund for retention and use of fees and revenues at district level since 1996), but these were managed by the district.

Many in the system saw RBF as a way of reviving the existing public system. Structures which existed but were not fully operational, often for resource reasons, were supported – for example, district supervision and the Health Centre Committees.

In summary, the adoption and adaption of RBF was strongly shaped by the historical legacy of Zimbabwe’s relatively high performing health system, but also its almost total collapse during the economic crisis, which started in the late 1990s and is on-going. These and the political relationships between the post-colonial Mugabe government and international partners influenced RBF directly, and also indirectly through ideological channels, which are discussed next.

#### Ideologies and values

Initial resistance to the RBF concept was fuelled in part by the public service ethos within the health sector, although this had been eroded during the crisis years. In particular, there was resistance to reforms which threatened equity, which had been a strong health policy thrust in Zimbabwe post-Independence.

As the programme became more embedded however, there was an appreciation of how it delivered financial resources to lower levels, though some report the health system to have remained very hierarchical with limited providers’ autonomy.

Over time, local actors have been exposed to and have in part absorbed the ideology and values of the RBF community, which emphasise ‘core principles’ of RBF, such as the separation of functions. Repeated exposure to SINA courses^1^ and strong RBF champions over the years has played a role in embedding RBF concepts in key leaders. However, debates about the ‘purity’ of the RBF model (based on the SINA-defined ideal) in Zimbabwe continue - in 2017, a MoHCC team reportedly analysed the ‘purity’ of their RBF model according to the SINA principles and concluded that it needed ‘purifying’. For example, risk-based verification, which was then under development in order to reduce management costs, was considered unacceptable. Equally, the planned institutionalisation of RBF within the Project Coordination Unit (a semi-independent unit set up in the MoHCC to manage Global Fund programmes) was considered insufficiently separate from the MoHCC ‘regulator’. The mix of verification and payment undertaken by Cordaid (and later Crown Agents) was also considered unacceptable, as was any form of pooled procurement. However, importantly, the variation in the RBF design in Zimbabwe can be seen as part of a successful adaptation to context, as highlighted above and by key informants.

*‘There was a big debate about the autonomy of health facilities. The Soeters model [as outlined in SINA courses] sees health facilities as stand-alone, not part of a system. But Zimbabwe sees health facilities as part of a system. We agreed to meet half-way: facilities can develop their own operational plans, but according to targets given by higher levels. They are also guided in how to use the funds’ (national KI)*

In summary, the RBF model that emerged in Zimbabwe had to manage two, somewhat competing, ideologies - one emphasising an integrated system, the other a system in which roles and functions are distinct and actors have contractual relationships with one another.

#### Framing of concept and its evolution over time

A proposal to the World Bank from the MoHCC for pay for performance in 2008 focused on the retention of staff and framed RBF under that angle, but it also highlighted the risks of market distortion which earlier schemes to retain specific groups of staff, or staff in specific districts (such as targeted allowances), had caused [27]. This aversion to distortion of the labour market led to a delay in the health worker incentive component within RBF; this only came in in 2013, and even then, some facilities chose not to pay individual health staff incentives but to use funds for collective goods, like food, at least initially.

*‘Any retention schemes should feed into the pool and cover approved health workers with a possibility of reviewing existing scales upwards for all health workers rather than a targeted approach that destroys the teams at various levels’* [27]

As well as recognising the need to address poor health indicators, the MoHCC required resources and had confidence that the health system would use them well, particularly if they were delivered to frontline providers. It was not anticipating the need for new approaches at this stage – not perceiving RBF as a health system reform approach, but rather seeking investment. As a consequence, the issue of resources has continued to be important in the way RBF is perceived locally – though with dropping budgets for RBF in 2017/18, concerns have been emerging.

‘*The main issue has been keeping funding high enough. The recent drop in earnings has been very high – from $10,000 per quarter to $2,000 per quarter for some. This is a big shock’* (national KI)

RBF was later presented and perceived as an enactment of the wider government Result-Based Management (RBM) programme, which had been launched in 2005, but never implemented due to lack of resources [28] . RBM aimed to make all ministries more results-driven and included, in theory, results-based budgeting, personnel management, monitoring and evaluation, and management information system. Although quite distinct from RBF as later practiced in the health sector, it provided an ideological justification for it – what one KI called ‘ideological retro-fitting’. RBF is now seen as testing the RBM approach, which is also of interest to other sectors.

For others, such as higher level staff conducting private practice, the perception was that RBF was no different to their medical insurance work.

In summary, RBF was framed in different ways at different stages, including as an approach to motivate staff, as a way of resourcing the sector, as part of a wider transformation to results-orientation in the public system, and as a minor variation on a familiar fee-for-service payment mechanism. These views reflected not just contextual factors, as discussed, but also the position of different actors, which we now turn to.

### Actors

In this section, we analyse the position of the various actors on the issue of RBF, their roles and power relations, how this influenced decision-making processes, as well as how actors’ positions on RBF have shifted over time.

#### Decision-making

The MoHCC is a key player for technical decisions on health, though politics overrides when high profile issues arise. The MoHCC initially proposed that the World Bank focused on financing of PHC services, including village health workers, and providing basic inputs to rural health centres and district hospitals [29]. There followed a period of internal debate before RBF moved ahead, which involved give and take by both sides – the MoHCC took on board the concept, but the World Bank also had to alter its design to fit the needs of Zimbabwe. This careful adaptation was reported to lead to better ownership by the MoHCC over time.

The Provincial Medical Directors were seen as a key interface between policy and practice. The RBF programme worked closely with them from the start. Convincing them was seen as key to getting buy-in from the MoHCC. RBF offered additional resources but its potential to shift control was also recognised.

‘*The districts and provinces were advocates for RBF but maybe because of the resources’* (national KI)

Development partners (apart from the World Bank) were initially sceptical, reflecting divided international opinions on RBF and seeing RBF as mainly a World Bank project. However, they were won over by early evidence (from RBF reports on service utilisation) that RBF facilities were performing better. The HTF was reportedly persuaded to join by a mix of MoHCC enthusiasm, and local and international evidence. There was however a feeling of over-hyping of evidence and a lack of objectivity in how early results were reported, according to some key informants. Although an impact evaluation was conducted [30], the expansion of the scheme did not wait for these results (expansion took place in 2012 and 2014, with impact evaluation results only shared in 2016). A key driver from the facility side was enthusiasm with the increase in funds which RBF facilities were earning, compared to districts which were still receiving fixed-rate inputs (pre-2014).

Later iterations of the RBF policy do reflect use of programme evidence – for example, the prices of indicators and their weights were adjusted over time to address areas of over- and under-performance, although these decisions are complex and so the role of external experts has been prominent in the decisions of the National RBF Steering Committee.

#### Roles and power relations

The National RBF Steering Committee (NSC) provides the overall leadership for RBF. It is co-chaired by the MoHCC (Principal Director, Preventive Services) and a lead development partner and includes the Ministry of Local Government (which manages all public infrastructure), civil society, Ministry of Finance, mission institutions and development partners. However, key informants expressed mixed views on the extent to which the MoHCC provided leadership in the committee. Some felt that it was mainly agreeing to changes which had been pre-prepared by external players.

*‘Ownership and leadership were there – it was chaired at a high level – but there was limited follow-through. In the early years, it depended on the World Bank and partners’ (national KI)*

Others highlight that Zimbabwe has exceptional ownership in the RBF process compared to other countries. The MoHCC clearly has veto power. However, the complexity of RBF design and management contributes to a technical ‘black box’ which some NSC members struggle to engage with.

*‘There is a pretence at participatory meetings, then they go back to hotel rooms and do things on their own … Results are presented in such a complex way that no-one can engage with them’* (national level KI)

Since its initiation, Cordaid provided the secretariat for the NSC, which was presumably more active early in the process, but by 2016, meetings were no longer very frequent, with the minutes referring to the ‘urgent need to kick start the RBF NSC’ [31]. The membership was also large, which may have been a factor in efficient and informed decision-making (with 38 people listed in minutes from 2016). According to key informants, it has been challenging to gather committed members, willing and able to attend regularly and spend time to study the issues scheduled for debate.

RBF is embedded in an MoHCC hierarchy which is well respected, with communications going from the MoHCC to Provincial Medical Directors, then DHEs.

‘*In the DRC [Democratic Republic of Congo], donors give instructions to facilities, but here systems are well established. This means that we are not running a parallel system. Implementers have to work closely with the Ministry of Health’* (national KI)

Districts were especially important, planning services and managing resources on behalf of PHUs. Under RBF, there was a potential for their purchasing and resource management role to be reduced, however, the MoHCC insisted that District Medical Officers needed to approve the operational plans drawn up by PHUs, and in practice, most respondents did not feel that RBF had introduced a large shift in the balance of power in the health system, but had rather helped to support traditional roles.

The governance of the RBF programme was linked to existing local governance structures, such as provincial and district development committees, and the District Health Team, which coordinates across sub-sectors within health.

Development partner roles increased post-crisis, focussed on preventing the collapse of the primary care system. In relation to RBF, there was an initial divergence between the HTF and the World Bank, but the HTF later (in 2014) agreed to fund the same model, with the encouragement of the MoHCC. The World Bank was seen as poor in communicating with other partners in the sector, and there have been continued problems of coordination and information sharing between partners in general.

Seen from other side, some development partners felt that stronger MoHCC leadership could have helped enforce greater harmonisation, including on data analysis and sharing.

There was some discomfort about international non-governmental bodies, such as the RBF implementation agencies, ‘verifying’ MoHCC work, which led to a more neutral language of ‘field officers’ being used for the Cordaid and later Crown Agents staff, who do indeed provide a wider range of roles than simple verification (also providing training, mentoring and wider support to facilities and districts).

A summary of the main actors and their positions on RBF over time is provided in Table 5. It appears that the adoption of RBF was predominantly influenced by senior staff in the MoHCC at national and provincial levels, in interaction with key partners. Thereafter the RBF Steering Committee was formally in charge, though sometimes more in a veto position than in the driving seat. RBF was supported by the existing governance structures but within a landscape marked by some tensions over coordination with, and between, development partners.

**Table 5:** Summary of key actors’ positions on RBF over time in Zimbabwe

Actors	Initial position on RBF	Evolution of position over time
Ministry of Health	Initial distrust and lack of knowledge about RBF	Key managers at national level take ownership, though residual concerns remain about it being another ‘vertical’ approach; resistance is also felt from programme managers uninvolved in RBF.
		Provincial Medical Directors and DHEs appreciate it as bringing supportive resources and tools
		PHUs gain relatively flexible resources, although are concerned about fall in budgets and intensive procurement procedures; hospitals have not benefited significantly to date though the policy is now being extended to district hospitals in all areas.
		Staff at PHUs benefited from incentives but have some concerns, especially over how they are distributed internally.
Ministry of Finance	Thought to be supportive of this as enabling an (adapted) trial of results based management	The MoF has supported RBF with some co-financing in the World Bank-supported districts; may be interested to extend to other sectors; however, the on-going resource squeeze is a major constraint.
Communities	No prior exposure	Communities have benefits from important inputs in the quality of care at PHU level, though effects on financial protection are not so clear.
		HCCs have gained from acquiring resources to manage; however, wider links with and accountability to communities continue to be limited.
Development partners	Most development partners initially perceive this as a World Bank project; some early resistance to the approach	Gradually won over by what seem to be promising early results; later support roll-out, though there are concerns about the model being ‘over-sold’ by a number of development partners. They see gains as the result of a wide range of system-supporting interventions which happened concomitantly.
		The World Bank is able to portray RBF as successful, although its own impact evaluation is more mixed.
Implementers	Cordaid had long-standing expertise on operating RBF and was an advocate. Crown Agents was initially less experienced.	Cordaid remains supportive of RBF and is supporting institutionalisation in its districts.
		Crown Agents has gained experience of RBF and continues to operate the policy in HDF-supported districts, with UNICEF continuing as fund manager.

### Distribution of resources

In this section, we examine how different stakeholders were affected by RBF, followed by its impact on health financing and ownership, on corruption and rent-seeking, and on equity of service delivery.

#### Gains from reform

In assessing which groups in the system have gained or lost from RBF, the main beneficiaries, as perceived by most KIs, have been the *PHUs*, which have gained in autonomy over some routine resource management, although this also comes with considerable restrictions and controls over how funds are spent – controls imposed both by the RBF model but also the national public financial management rules.

In terms of amounts of funding received, PHUs with larger catchment areas have benefited – they previously received $750 per quarter from the HTF, whereas after RBF, amounts ranged from $700 to $4000 per quarter in one district visited by the research team, and were mainly dependent on population size, which varied substantially from 1000 to 15,000 per health centre.

*‘Low performance goes hand-in-hand with low catchment populations’ (local KI)*

There were concerns about the drop in prices for indicators in 2017. Facilities were concerned that they would no longer be able to manage with these lower prices, given continuing limited support from the central budget.

*Hospitals* in most districts (in the 42 Crown Agents/HDF-supported districts) did not receive RBF payments before 2018, and by contrast with PHUs were in a very poor financial and material state [22]. RBF hospitals (in Cordaid/World Bank-supported districts) on average received 136% more revenue per quarter than non-RBF hospitals [22]. District hospitals in HDF districts were therefore looking forward to moving to an RBF-based system in 2018, although their expectations remained dependent on adequacy of the RBF budget.

A condition for receiving RBF funds was the removal of fees for maternal and child care, and this caused problems for some sub-sectors such as the *Rural District Councils*, which used to collect revenues from their health facilities. However, most had agreed in the end to remove them in order to join the scheme.

*Staff at PHUs* have generally benefited from having the funds and materials to work with, as well as bonuses. This was reflected in the impact evaluation, which found higher levels of job satisfaction and autonomy in RBF facilities, compared with non-RBF facilities [30].

*‘The work environment improvement and ability to make decisions at their level is what motivated health staff. Rural health workers were also more cognisant of the work they had to do’ (local KI)*

However, there were concerns about their distribution. The distribution of the 25% of RBF payments to staff follow hierarchy and attendance and covers all staff, whether working on maternal and child health services or not. This aims to reinforce teamwork but caused some resentment – for example, the Environmental Health Practitioners receive bonuses, but many of their colleagues do not perceive their contribution as they are not facility-based. Some facilities also informally pass on benefits to Village Health Workers who bring in clients.

While RBF facilities reported significantly higher autonomy in financial allocation, there was less difference in autonomy on allocating tasks to staff [30]. Staff in RBF facilities were not any more satisfied with career development factors, such as opportunities for promotion, than staff in non-RBF facilities. In addition, staff in RBF facilities had low motivation, mainly because of issues which are largely unrelated to RBF, such as teamwork, the work environment, recognition and the leadership of the facility. They expressed their dissatisfaction with the relative proportion of incentives for their tasks and slow disbursements. Higher workload and consequent burnout was also a source of dissatisfaction [30]. The evaluation also found that staff were demotivated because DHE supervision was focused on fault finding and lacked confidentiality.

From the *patients’ perspective*, RBF contributed to greater drug and staff availability, as well as infrastructure improvements (such as mother’s waiting homes, which many facilities built using RBF funds, with wider community support). Client satisfaction with antenatal services in RBF facilities improved with regard to the respectfulness of health staff in the World Bank evaluation; however, the same improvement was also observed in the control (non-RBF) facilities [30]. The amount of time clients spent waiting to be seen was reasonable in the RBF facilities, better than in the control facilities, and trust in the skills and abilities of the health workers’ also improved. Fees for maternal and child health care have been reduced [32] and mother and child health indicators have improved from their low point during the crisis [33]. However, it is important to recognise that this is the result of a wide range of donor support for reproductive, maternal and health and primary health care systems over the period, not just RBF.

RBF also strengthened the role of the *Health Centre Committees*, enabling a switch in focus from resource mobilisation to resource allocation [34], though many challenges remain in developing community awareness and engagement and ensuring the effectiveness and sustainability of Health Centre Committees [30, 32, 35].

Divergent views were expressed on the effects on *higher level managers* in the health system – some felt that they had lost control but others that they had gained more strategic oversight.

Large programme managers in the MoHCC continued to purchase independently and had not been included in the RBF approach in any case. For those who have taken a more central role in the oversight of RBF, there have been many intangible pay-offs in the form of attendances at RBF trainings, study tours and conferences, with the prestige of Zimbabwe being presented as a high performer in the RBF world (in 2016, the global RBF workshop was hosted in Harare, with an opportunity to show case the country’s experience).

For *provincial and district level managers*, RBF provided some resources to support their work but the payments did not link very closely to performance, being more linked to routine activities to support the programme.

‘*How motivating is it for DHEs and PHEs? There is lots of work but limited reward. So motivation needs to be intrinsic – wanting to be able to present good results in provincial review meetings, for example’* (national KI)

The *Ministry of Finance* (MoF) was said to be a key champion from the start, perhaps because RBF allowed for some level of testing of the RBM concept, previously introduced but not implemented due to lack of resources. This support was signalled by government co-funding, which started with $1 m in 2015. This increased to $10 million in the 2018 budget. However, the MoF was reported to be sceptical of the staff incentives component, which is not practiced in other sectors.

‘*It took a while to get the funds but was seen as a signal. The MoH struggles to get any money from the MoF*!’ (national KI)

From the *World Bank* perspective, Zimbabwe provided a rapidly scaled up and relatively well managed RBF programme as a demonstration model, although with the shift from the Health Results Innovation Trust Fund to the Global Financing Facility, the conditionality linked to results-based approaches appears to have softened. As a national KI commented, ‘*It may be possible to move away from the blueprint now*’.

*Other international agencies* have also benefited from RBF, including the fund manager for the HDF scheme, UNICEF, which plays multiple roles, including drug procurement on behalf of donors. There is some resentment locally about this by-passing of government systems, and complaints that there is no transparency on overhead costs charged by UN agencies.

‘*No multi- or bilateral donors could give [funds] to the state – that led to the Republic of UNICEF’* (national KI)

Other important players include the *implementation agencies* – Cordaid and Crown Agents – which were contracted to manage RBF in 18 and 42 districts respectively. The MoHCC was initially distrustful of Cordaid but came to appreciate their support. Crown Agents was later able to benefit from Cordaid’s greater prior expertise in RBF and the two organisations have cooperated well in general, sharing one project implementation manual, though with some tensions and difference in verification/counter-verification systems [32] and some duplication (separate working groups on information systems, for example).

In summary, RBF has brought some degree of benefits to most parties, though few gains are unambiguous. For example, while front line providers gained, they were also subject to increased demands, as was also the case for staff.

#### Impact on ownership structure and financing

In this section we examine whether and how RBF has influenced ownership structures and financing flows for health care in Zimbabwe. RBF channeled funding to the public and mission PHUs and some first level hospitals, but that this has not changed ownership structures within the health sector, with the wider health market more affected by economic changes, such as reduction in municipal levies and challenges to the private insurance market (see Table 4).

The original RBF scheme was budgeted at $2 per capita [36] and a recent study estimated RBF’s incremental cost at $3.19 per capita [37]. Overall RBF expenditure in 2015 across 60 districts was just over $18 million [22], while the current annual budget for RBF across 60 districts is $22 million. In comparison with an estimated $69 per capita public health spend [22] (including external aid but excluding out of pocket payments) and overall health needs, RBF’s contribution looks small. However, compared to public funding arriving at local levels, the amounts are seen as significant by local actors.

‘*The limited funding from the Government of Zimbabwe is one of the main challenges. Health facilities are 100% dependent on RBF’ (local KI)*

There is no overall data on how RBF revenues were used by facilities, but the impression of many key informants is that spending was initially quite focused on infrastructure, including maternity waiting homes. Expenditure on drugs has also been a main component, partly because of problems in the national drugs supply, which recent reforms of pharmaceutical supply and additional funds from the Health Levy are meant to be addressing [22]. Other expenditures include medical equipment, utilities, travel and subsistence (including to collect quotations for procurement by the in-charges or Health Centre Committee members). Some facilities were reported to spend up to 20% of their RBF funds on travel and subsistence, which is seen as excessive – the MoHCC sent a guidance note in 2018 limiting this to a maximum of 10% of RBF revenues.

From the HTF side, the funding was not additional, in that resources were already committed to supporting the primary care system before switching to RBF in 2014. However, given restrictions on the Health Results Innovation Trust Fund, it would be reasonable to regard the World Bank funding as additional.

Overall, then RBF provided small but significant resources for health care, which still fell far short of needs even at primary care level.

#### Corruption and rent-seeking

The general perception of key informants was that corruption in Zimbabwe was a high-level phenomenon, not one which pervaded the system, not least because of the limited resource flows through it (with the bulk of procurement handled by external agents).

RBF offered an attractive financing modality in the context of lack of trust in government, channelling funds into lower levels of the system. At the start there was concern that this might increase corruption at PHU level. However, this is perceived not to have occurred, largely due to tight controls and also residual professionalism in the system. Most key informants felt that the main risk of RBF was rather inefficiency, in terms of additional time required for procurement, purchasing and reporting by in-charges, who are not only the manager but also the main clinical person at clinic level. A further issue was uncompetitive prices for items like pharmaceuticals, if clinics are purchasing in small quantities, rather than getting bulk procurement at a higher level [22]. Lack of cash in the overall economy was another factor delaying disbursements, peaking in 2017, though this has now been mitigated by increased use of ‘plastic money’.

‘*The day after being paid their subsidies, suppliers would go to the clinics with three quotes, all on different letterheads, with theirs the cheapest! But we picked this up’* (national KI)

More generally, deeply rooted corruption remains a challenge and is seen as having deteriorated over the period because of the dire circumstances faced by the country.

#### Impact on equity of service delivery

There has been a focus on creating an equitable health system post-independence in Zimbabwe, despite wealth inequalities. However, the economic crisis of the 2000s led to widespread exclusion and increased financial barriers. The 2013 Constitution commits Zimbabwe to ensuring access to all for essential health care, though with the proviso of it being within the limits of the resources available to it [38]. RBF has contributed to an overall return to health system functionality, alongside other development partner resources post-crisis.

*‘RBF helped to accelerate Zimbabwe to get closer to where it had been a decade earlier’ (national KI)*

In relation to equity, RBF has an inherently equitable design, in that it focuses on maternal and child health outputs at primary level in rural areas, whose users are more likely to be disadvantaged. The programme also incorporates a remoteness bonus, although this is low in practice, and most of the RBF revenues have been driven by quantitative outputs, which largely reflect catchment populations. This disadvantages more remote areas, which typically have smaller catchment populations [22].

There were some indications of greater benefit to poorer households in the 18 districts [30]. However, the impact on financial barriers (changes to household out of pocket payments) was not reported in the impact evaluation, despite removal of user fees being a key goal of RBF. In general, inequalities of access at the population level remain^38^, with institutional delivery rates, for example, roughly twice as high (90%) for the top quintiles as the bottom (46%) [24].

## Discussion

This study is the first to our knowledge to focus on the political economy of RBF, particularly in relation to how it alters distribution of resources in the health sector. It has examined the case of Zimbabwe, one of the few countries in Africa to have scaled up RBF nationally and which has been neglected in published academic literature to date. It adds to the limited published evidence on political economy of health financing reforms in low income countries [6, 8, 9, 39], with even less attention having been paid to FCAS settings, where by definition institutions are weaker and liable to be even more vulnerable to power and resource imbalances. In this case study, characteristics of fragility, such as dependence on external resources and actors, are exhibited, alongside atypical features in fragile settings, such as institutional capacity to resist or at least reform externally introduced programmes.

In relation to our first question, on adoption, our findings match some of other recent studies in the regions which look at RBF policy adoption in highlighting the important role of donors, in terms of offering financial and ideational inducements [9, 11], and also how crisis – in this case, economic and political – was an important trigger. This is not unique to RBF – external players have been influential in previous health financing reforms [40], such as user fee removal, although in that policy, domestic actors were arguably much more dominant in adoption [41]. Contrasting with a recent set of studies on RBF scale-up, the pilot phase did not play an important role and there was limited influence of local policy entrepreneurs [10]. In Zimbabwe, we also document the ideological legacy of the post-colonial period and residual resources within the health system, despite more than a decade of severe under-financing, and how that contributed to modifying and adapting RBF to what MoHCC staff saw as their own unique context, in particular wanting to maintain a coherent system. This led to more integrated implementation and genuine ownership over time, despite initial resistance to the RBF concept, at least amongst some key technical staff in the MoHCC, which may explain its rapid scale up and enable its sustainability [10, 42] . Other countries which have lacked the capacity to push back on donor plans and to ensure aligned policies, such as Sierra Leone, have seen more start-stop approaches to RBF [43]. A review of the use of RBF in humanitarian settings also highlights the need for adaptability to these very challenging contexts [39].

The contextual factors were key in the uptake of RBF in Zimbabwe, especially the fiscal constraints. RBF was initially seen as important to address staff retention and to address poor indicators, providing revenue to revitalise the health system. It was not conceived as a health system reform or as a needed incentivisation from the MoHCC side, but as funding was conditional on RBF mechanisms, this was accepted. Later on, some came to appreciate, however, that RBF was able to channel resources effectively to frontline providers and also to provide more complete data on results [32]. There was also a later retro-fitting of RBF into the RBM government programme, which provided ideological coherence to deployment of RBF in the health sector.

As documented in other settings [8, 9, 12], scientific evidence was not the main driver of scale up of RBF: from the MoHCC side, there was no demand for robust evidence prior to scale up as there was confidence that any resource injection would be effective; from the funder side, there was a perceived bias towards positive results. In any case, scale up preceded sharing of the impact evaluation. We note however that this is not unique to RBF: a recent comparative analysis of learning across low income health systems found that the choice and application of evidence is often “purpose-driven” and predefined by political agendas [42].

In relation to our second question on implementation, the MoHCC retained authority over decision-making, however, the complexity of RBF programmes poses a challenge, with our study and a related study of the effects of RBF on strategic purchasing highlighting the risk of important technical decisions being decided in smaller groups and merely being ratified by governing bodies [44]. The discussion about ‘purity’ of the model also raises the issue of trade-marking of concepts: while some groups have historically had a very strong influence over the development of RBF in low and middle income countries, it is clear that there are differing understandings and applications across settings and indeed that some degree of local adaptation may be essential for the schemes’ success [8, 39] and to ensure that RBF is not seen as a standalone programme.

In terms of impact on distribution of resources (our third question), RBF, like any health financing reform, does involves a shift in power and resources, but the Zimbabwe case study does not suggest a major change, perhaps because the health system was relatively well developed prior to the crisis and retained many of its structures and relationships. There was some pay-off from RBF for those in top positions – international prestige of presenting in international meetings, for example – and equally, resistance from others who were outside this group (for example, managers working on programmes with different funding modalities). This corresponds to the notion of ‘pay for participation’ [12] whereby insider elites are co-opted into support of policies. International agencies which were contracted to operate RBF also gained from developing expertise in RBF.

Most agreed that frontline facilities gained from increased resources, although there appears to be a paradox at the heart of RBF, as it simultaneously passes resources and (potentially) control over those resources to the periphery, while also using them as a way of establishing control (using contracts, reporting, verification and sanctions) to direct behaviour. In relation to staff, the picture is mixed, which reflects wider literature [45–47], with some clear benefits in terms of funds to invest in working conditions but also concerns in relation to equity of payments (payment by seniority, rather than performance) and effects on workload. Similarly, the impact on equity of services is mixed, with the package of care potentially favouring lower income households but the focus on volume of services favouring facilities serving more populated (typically less remote) areas. This is again a feature shared by many other RBF programmes in Africa and supports the argument that more focus on equity analysis of RBF is required [48].

In terms of the resources which RBF brought to the under-financed health system in Zimbabwe, this represented a small but significant (and partially additional) increment in public resources for health, around 3% of the estimated need per capita for an essential health care package or 5% of available funding. Rather than providing an incentive, due to shortfall in the public budget it was used as the main source of funding for non-salary recurrent costs at PHU level, thus functioning as a core financing mechanism [44] and supporting the improved performance of the sector as a whole [30, 32]. Its future role within the wider health financing landscape remains unclear.

In relation to limitations of this study, our KI sample was large but not comprehensive (partly because of field work time but also due to the movement of participants into new and less accessible posts). However, care was taken to include most of the main stakeholders (people holding relevant positions in the Ministry, development partners, implementers and technical support roles) in this policy, not just present but over its history. In some cases, KI were time-limited so interviews had to focus on a limited range of questions, also to reflect their period of engagement with the policy. We also have to bear in mind that stakeholders had specific positions, reflecting institutional and personal interests, which we took into account during analysis. Equally, many of the documents which describe the process of policy development and roll out are confidential or not available, so while the researchers tried to access as broad a range of documents as possible, they could not be comprehensive. Finally, we note that this represents only one case study, so any generalisation has to be cautious while the body of evidence is built. Other countries will need to examine whether their contextual factors are similar to Zimbabwe’s in ways which make similar outcomes likely.

The article also sheds light on how political economy analysis may need to be adapted to be usefully applied to FCAS settings. While political economy in higher income settings often focuses on the role of politics, bureaucratic factions, interest groups and beneficiary organisations in influencing policy development and outcomes [49], in the Zimbabwe context, these groups are less organised and influential, with individual leadership, donor positions and small amounts of marginal resource having disproportionate influence. Participation by the population is typically weak [30]. Organisations which can bring technical and financial capacity to bear, for example by supporting implementation, can play a very significant role in the emergence and development of reforms. Although these findings are specific to Zimbabwe, they are likely to also apply in other low-income settings - for example, fragile and conflict-affected settings - although each presents a unique historical case study, requiring careful analysis. The specific configuration of ideological legacies from Zimbabwe’s recent independence, for example (emphasising equity goals), combine in this case with a health system residual capacity (which can resist externally imposed models but can also deliver results) and a current financial dependency on external resources to explain the patterns of adoption, adaption and impact identified.

## Conclusion

This study highlights resource-seeking motivations for adopting RBF in some low and middle income settings, especially fragile ones, but also the potential for local health system actors to shape and adapt RBF to suit their needs in some circumstances, where sufficient technical capacity and institutional self-confidence exists. While this means less structural disruption in the health system, it increases the likelihood of an integrated approach and sustainability, though resources remain a key constraint in Zimbabwe, as in many health systems. We highlight the mix of autonomy and control which RBF can bring for frontline providers and argue for clearer understanding of the role that RBF commonly plays in these settings – although it is portrayed as an incentive approach, it is functioning more as the main provider payment mechanism for under-funded primary care. Donor organisations and governments need to clarify its role within the overall health financing architecture.

**Table:** Box 1 Looking ahead to institutionalisation

In 2018, an institutionalisation process started, with the 18 World Bank/Cordaid districts moving from external management to management by the Project Coordination Unit in the MoHCC – what one informant called ‘*baby steps towards re-establishing strategic purchasing in the MoH’* (national KI). During the initial period, staff were transferred from Cordaid, to retain their expertise, and posts were externally financed. The unit will be semi-independent within the MoHCC, reporting to the Permanent Secretaries of the MoHCC and Ministry of Finance. This will constitute a transition to internalisation of RBF, with the aim of full transition by 2020. It is clearly not evident as yet how well this will support performance pressures and ensure regular payments to facilities, and many questions remain open about local level RBF structures in future and whether or how the intensive role of the field officers (in training, support, verification, follow up and mentoring) will be replaced. Some have also noted the need for a stronger central performance management unit in the MoHCC, which could have oversight over RBF and also ensure its fuller integration. One crucial factor is whether the government is able to take over the financing commitment for RBF, which currently has limited financial security, with core funding from the World Bank stopping in 2018 and the HDF under-financed. Ultimately greater Government of Zimbabwe funding is needed for full ownership of RBF. In principle, RBF could be extended to cover a full package of basic services – absorbing indicators beyond reproductive, maternal and child. In 2017 a few indicators on tuberculosis and antiretroviral therapy were added, but with extremely low payments ($0.05 per TB case detection, for example). Movement in this direction is currently dependent on other donors ‘buying in’ to RBF to virtually pool funds and purchase additional services which are delivered at PHUs and district hospitals. Ideally, these priorities would include more local iteration to reflect disease burdens. The current indicator list and prices are nationally determined. Longer term, a consensus needs to be reached on whether RBF’s main function is to incentivise under-performing areas (i.e. providing a small, targeted financing component) or to be the main channel for funding non-salary recurrent costs at facility level. This remains unclear in the draft National Health Care Financing Strategy [50] and key informants also held divergent views on this. Most favoured a mixed payments system, with base inputs for salaries, centrally procured drugs operating on a pull system and RBF as an incentive only (in which case it could reduce from $20–25 million per annum to an estimated $5 million). The right level of incentives is unclear, but it could potentially be 10% of salaries, with a higher level for managers [22]. At present it is covering some of the core recurrent costs, but without better cost information, it is not clear which or how much. It is simply providing additional resources, which are gratefully received. The development of a national health insurance system, if it occurs within the medium term (and bearing in mind the challenges posed by the current low formal employment rate), will also need to articulate with RBF, possibly focusing on addressing affordability while RBF incentivises the supply side for neglected services.	

## Data Availability

The datasets generated and analysed during the current study are not publicly available due to protection of confidentiality of participants (given the difficulty of fully anonymising qualitative transcripts).

## Abbreviations

DHE: District Health Executive
FCAS: Fragile and conflict-affected states
HTF/HDF: Health Transition Fund, later Health Development Fund
KI: Key informants
MoF: Ministry of Finance
MoHCC: Ministry of Health and Child Care
NSC: National Steering Committee
PHU: Primary Health Unit
RBF: Results-based financing
RBM: Results-based management
UNICEF: UN Children’s Fund

## Footnotes

1 SINA runs training courses and consultancies in RBF design and management. It is based on very specific design principles, which define a ‘pure’ RBF model. http://www.sina-health.com/
